# La Grippe

**Published:** 1891-05

**Authors:** 


### HALL'S JOURNAL OF HEALTH

TRUTH DEMANDS NO SACRIFICE; ERROR CAN MAKE NONE.

Vol. 38. MAY, 1891. No. 5.

### LA GRIPPE.

This fearful epidemic has played sad havoc the past few weeks, in various sections of the country, New York, Chicago, Pittsburgh, and our sister city of Brooklyn having suffered most keenly from its ravages. The death rate of these populous centres, for the most part widely distant from each other, has been something fearful, reminding one of the worst days of the Asiatic cholera, nearly half a century ago.

To raw spring weather, which continued with scarcely an interruption, to the middle of April, may be largely attributed the prevalence of this unusually fatal malady, whose attack in the primary stage so nearly resembles an ordinary influenza as to give little apprehension of its dangerous character. We confess to but scanty knowledge of this strange malady. It would seem to make its main attack upon the weaker and more impaired organs or members of the afflicted. The brain, heart, lungs, liver, kidneys do not escape, if suffering from any chronic weakness. Sometimes the point of attack is in the joints of the limbs, accompanied with severe pains and inordinate weakness. Then again the whole nervous system is assailed. Its ravages are so swift that death is oftentimes near without being suspected. The wisest treatment is found to be careful home nursing, under the direction of a skillful physician, with sufficient foresight to assist nature in her efforts to overcome disease without paralyzing her energies by a too free use of drugs.

The primary cause of this prevailing epidemic is believed to be atmospheric ; that we are now being subject to an invisible army of its health-destroying germs that enter the system with the air we breathe, and poison it, sometimes beyond recovery, before the evil becomes manifest. The inhalation of camphorated air by a simple arrangement of air tubes in a bottle of the solution will be found to be an excellent preventive of various infectious diseases, including La Grippe.

